# Acoustic Radiation Force Impulse Imaging for Noninvasive Evaluation of Renal Parenchyma Elasticity: Preliminary Findings

**DOI:** 10.1371/journal.pone.0068925

**Published:** 2013-07-11

**Authors:** Le-Hang Guo, Hui-Xiong Xu, Hui-Jun Fu, Ai Peng, Yi-Feng Zhang, Lin-Na Liu

**Affiliations:** 1 Department of Medical Ultrasound, Shanghai Tenth People’s Hospital, Tenth People’s Hospital of Tongji University, Shanghai, China; 2 Department of Nephrology, Shanghai Tenth People’s Hospital, Tenth People’s Hospital of Tongji University, Shanghai, China; University of Louisville, United States of America

## Abstract

**Objective:**

To evaluate the diagnostic value of acoustic radiation force impulse (ARFI) to test the elasticity of renal parenchyma by measuring the shear wave velocity (SWV) which might be used to detect chronic kidney disease (CKD).

**Methods:**

327 healthy volunteers and 64 CKD patients were enrolled in the study. The potential influencing factors and measurement reproducibility were evaluated in the healthy volunteers. Correlations between SWV and laboratory tests were analyzed in CKD patients.?Receiver-operating characteristic curve (ROC) analyses were performed to assess the diagnostic performance of ARFI.

**Results:**

The SWV of healthy volunteers correlated significantly to age (r = −0.22, P<0.001, n = 327) and differed significantly between men and women (2.06±0.48 m/s vs. 2.2±0.52 m/s, P = 0.018, n = 327). However, it did not correlate significantly to height, weight, body mass index, waistline, kidney dimension and the depth for SWV measurement (n = 30). Inter- and intraobserver agreement expressed as intraclass coefficient correlation were 0.64 (95% CI: 0.13 to 0.82, P = 0.011) and 0.6 (95% CI: 0.31 to 0.81, P = 0.001) (n = 40). The mean SWV in healthy volunteers was 2.15±0.51 m/s, while was 1.81±0.43 m/s, 1.79±0.29 m/s, 1.81±0.44 m/s, 1.64±0.55 m/s, and 1.36±0.17 m/s for stage 1, 2, 3, 4 and 5 in CKD patients respectively. The SWV was significantly higher for healthy volunteers compared with each stage in CKD patients. ARFI could not predict the different stages of CKD except stage 5. In CKD patients, SWV correlated to e-GFR (r = 0.3, P = 0.018), to urea nitrogen (r =  −0.3, P = 0.016), and to creatinine (r =  −0.41, P = 0.001). ROC analyses indicated that the area under the ROC curve was 0.752 (95% CI: 0.704 to 0.797) (P<0.001). The cut-off value for predicting CKD was 1.88 m/s (sensitivity 71.87% and specificity 69.69%).

**Conclusion:**

ARFI may be a potentially useful tool in detecting CKD.

## Introduction

Chronic kidney disease (CKD) is a major public health problem in developed countries [1]. In the United States, there is a rising incidence and prevalence of kidney failure, which has poor outcomes and high cost. Data from United States renal data system 2000 Annual Data Report suggests that the incidence and prevalence of end-stage renal disease (ESRD) have doubled in the past 10 years and are expected to continue to rise steadily in the future. Through its effect on cardiovascular risk and outcomes as well as ESRD, CKD directly affects the global burden of death caused by cardiovascular disease, the most common cause of premature morbidity and mortality worldwide [2]. Also, CKD is highly prevalent in developing countries [3]. In China, the prevalence is as high as 10.8% [4].

Regardless of etiology, all patients with CKD show a progressive decline in renal function with time. The process is largely irreversible, inevitably leading to ESRD, a condition that requires life-long dialysis or renal transplantation. Histologically, CKD manifests itself as progressive glomerulosclerosis, vascular sclerosis, and tubulointerstitial injury, which encompasses tubular atrophy and interstitial fibrosis. If these histological changes associate with elasticity of renal parenchyma, there may be a new way to detect CKD through assessing the parenchyma elasticity.

Acoustic radiation force impulse (ARFI) is a newly developed, noninvasive, inexpensive, safe and convenient technique to assess the tissue elasticity. It is integrated in a conventional ultrasound (US) machine and is able to quantify the parenchyma elasticity by measuring the shear wave velocity (SWV). ARFI uses acoustic radiation force to transiently deform soft tissues in the region of interest (ROI), and the dynamic displacement response of those tissues is measured ultrasonically and is used to estimate the tissue’s mechanical properties [5]. It provides numerical measurements of tissue elasticity, softer (elastic) tissues are displaced further than stiffer (nonelastic) tissues for a given force magnitude. The stiffer a tissue is, the greater is the SWV [6]. SWV has been mainly evaluated in hepatic fibrosis and cirrhosis and has a good positive correlation with the grade of hepatic fibrosis [7], [8], [9]. In addition, ARFI has been used in the assessment of other organs, such as breast, prostate, testis and thyroid [10], [11], [12], [13].

ARFI has also been used to detect renal transplant’s elasticity. Chronic allograft nephropathy is a major cause of renal transplant failure, which is characterized by interstitial fibrosis and tubular atrophy [14]. Stock *et al*. [15] described a significant correlation between SWV and renal allograft fibrosis, and suggested that SWV may have potential for evaluating the grade of fibrosis in renal transplant. Therefore, we hypothesized that the change in renal parenchyma stiffness indicated by ARFI might be a useful sign for detecting CKD. This study was aimed to evaluate the potential diagnostic value of using ARFI to test SWV of renal parenchyma, which might be used as a “marker” for detecting and classifying CKD. Potential influencing factors and the measurement reproducibility were also analyzed.

## Materials and Methods

### Healthy Volunteers and CKD Patients

Between March 2012 and August 2012, 327 healthy volunteers (197 women and 130 men; age range, 17–87 years; mean age ± SD, 43.44±20.24 years) for health examinations were assessed with ARFI. None of them had abnormal renal function test (serum urea nitrogen, creatinine, uric acid, and urinary albumin) and abnormal imaging findings by conventional US such as cysts, stones, masses, nephrarctia or hydronephrosis.

We evaluated the influence of age and gender to ARFI measurement of the renal parenchyma in all 327 healthy volunteers, and chose 30 volunteers (16 women and 14 men; age range, 21–79 years; mean age ± SD, 43.37±20.19 years) randomly from the 327 healthy volunteers to evaluate other possible influencing factors including waistline, height, weight, body mass index (BMI), kidney dimension, and the depth for SWV measurements.

To test the inter- and intra-observer agreement, another 40 volunteers (16 women and 24 men; age range, 21–60 years; mean age ± SD, 38.53±12.21 years) were chosen from the 327 healthy volunteers randomly. When investigating the interobserver agreement, they were examined on the same day by observer 1 and observer 2, both of whom had previous training in performing ARFI and were blinded to the clinical and laboratory data. When investigating the intraobserver agreement, each volunteer was examined by observer1 twice with one day interval under the same conditions.

In addition, 64 CKD patient**s** (27 women and 37 men; age range, 23–89 years; mean age ± SD, 64.72±14.33 years) were assessed with ARFI. Serum cystatin C, urea nitrogen, creatinine, uric acid, cholesterol, triglycerides, low-density lipoprotein, high-density lipoprotein, calcium, phosphate, hemoglobin, albumin and urinary albumin were measured after an overnight fast of at least 10 h in CKD patients.

Estimated glomerular filtration rate (eGFR) was calculated with an equation developed by adaptation of the Modification of Diet in Renal Disease equation:
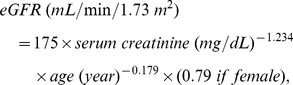
which is on the basis of data from Chinese CKD patients [16]. Diagnosis of CKD was on the basis of kidney damage or eGFR (ml/min per 1.73 m^2^), and was classified into stages as shown in Table 1. Clinical history and laboratory tests were used to make the primary diagnosis.

**Table 1:** Definition and stages of CKD*.

stage	description	GFR, ml/min/1.73 m^2^	n
1	Kidney damage with normal or increased GFR	≥90	11
2	kidney damage with mild increased GFR	60–89	11
3	Moderately decreased GFR	30–59	20
4	Severely decreased GFR	15–29	10
5	Kidney failure	<15 (or dialysis)	12

* CKD is defined as either kidney damage or GFR less than 60 ml/min per 1.73 m^2^ for 3 or more months. Kidney damage is defined as pathologic abnormalities or markers of damage, including abnormalities in blood or urine tests or imaging studies. In our study, CKD was defined as eGFR<60 ml/min/1.73 m^2^ or albuminuria. Albuminuria was defied as a urinary albumin.

The study was approved by the Ethical Committee of the Tenth People’s Hospital of Tongji University. According to the local legislation, oral informed consent was obtained from all healthy volunteers and CKD patients older than 18 years old, whereas for the healthy volunteers or CKD patients under the age of 18, oral informed consent was obtained from the next of kin, caretakers, or guardians on the behalf of the minors participants. The committee approved the consent procedures because the technique used in this study was incorporated in a commercially available US machine and its safety has been well documented. The study would not do harm and invasiveness to the healthy volunteers and CKD patients. The consent process was documented in a separate file after the oral informed consent was obtained.

### ARFI Elastography

All US examinations were performed using an Acuson S2000 ultrasound system (Siemens Medical Solutions, Mountain View, CA, USA), equipped with the ARFI function. The convex probes (4 C1, frequency range: 1–4 MHz) and mechanical index of 1.7 were applied. Tissue harmonic imaging was used to optimize the US images. ARFI was performed with the identification of target ROI (box with fixed dimensions of 1 cm in length and 0.6 cm in width; maximum depth of 8 cm) on a conventional US. To quantify the wave propagation speed, the quantitative implementation of ARFI, known as virtual touch tissue quantification was used.

**Figure 1:**
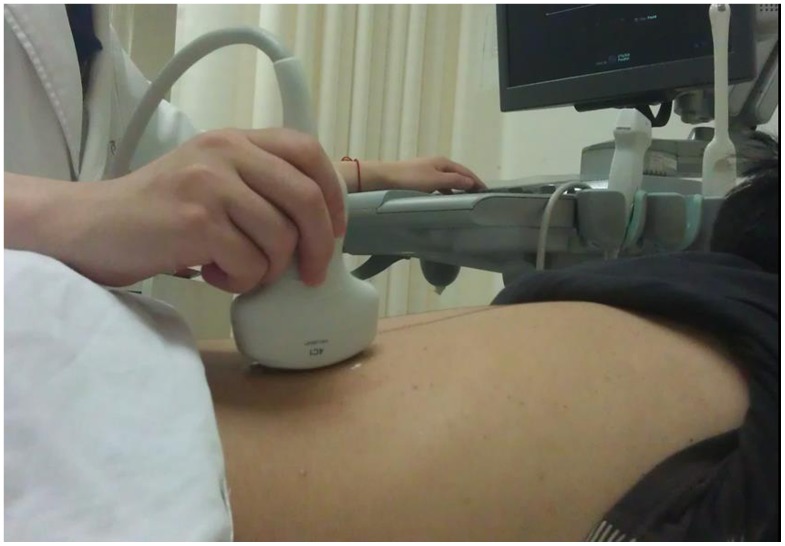
Volunteer underwent ARFI in prone position. To avoid compression, the probe was contacted with body surface without pressure.

**Figure 2:**
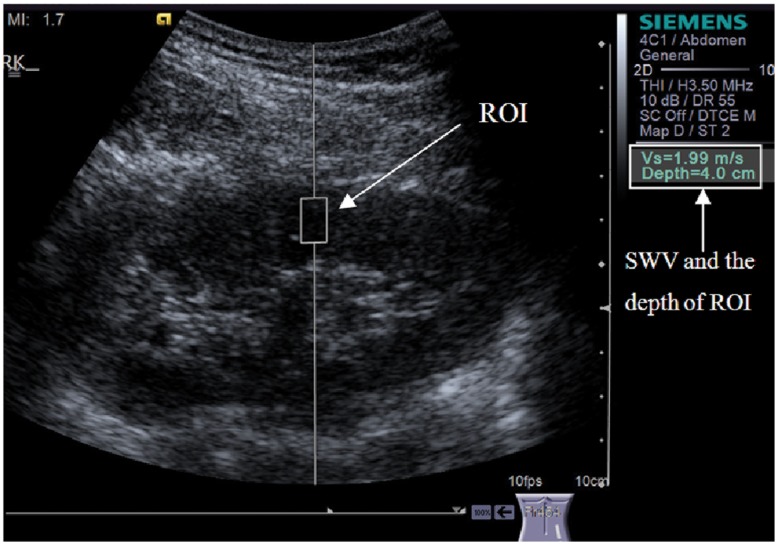
Measurement of SWV in the middle third of the renal parenchyma.

All cases underwent ARFI in prone position (Figure 1.) by observer1. When evaluating the interobserver agreement, volunteers were examined by observer1 and observer2. Before ARFI was performed, kidneys were checked by conventional US to avoid stones, cysts or masses. During real-time conventional US, the ROI cursor was moved onto the middle third of right renal parenchyma, excluding renal sinus and capsule (Figure 2.). Each observer measured 7 valid SWV measurements, with the maximum and minimum measurements been omitted. The mean of the remaining 5 SWV measurements, expressed in meters per second, provided numerical measurement that gives quantitative information about parenchyma elasticity property.

In the event of a non valid measurement (expressed as X.XX m/s), a repeated measurement was carried out. All measurements were performed during breath holding.

### Statistical Analysis

The SPSS version 17.0 software package for Windows (SPSS Inc, Chicago, IL) was used for statistical analysis. The continuous data were expressed as mean± SD (range). Influencing factors were analyzed with Pearson’s correlation coefficients. Inter- and intraobserver reproducibility were analyzed with intraclass correlation coefficient (ICC). Agreement was classified as poor (ICC = 0.00 to 0.20), fair to good (ICC = 0.40 to 0.75) or excellent (ICC = 0.75). A Bland-Altman plot was used to analyze observer-related variations. Unpaired *t*-test was used to analyze the differences in SWV between two different groups. The differences in SWV in different groups (327 healthy volunteers vs. 5 stages in 64 CKD patients) were evaluated by a one-way analysis of variance (ANOVA) test. When differences among them were found to be statistically significant (*P*<0.05), each group was compared with every other group using least significant difference (LSD) test. Correlation between SWV and variables (such as eGFR, serum urea nitrogen, creatinine and et al.) were analyzed with Pearson’s correlation coefficients in the 64 CKD patients. The diagnostic performance of ARFI in determining CKD was assessed using receiver operating characteristic (ROC) curves. The *P<*0.05 was defined as statistically significant.

## Results

### 1. Measurement Reproducibility (n = 40, from the 327 Healthy Volunteers)

**Figure 3:**
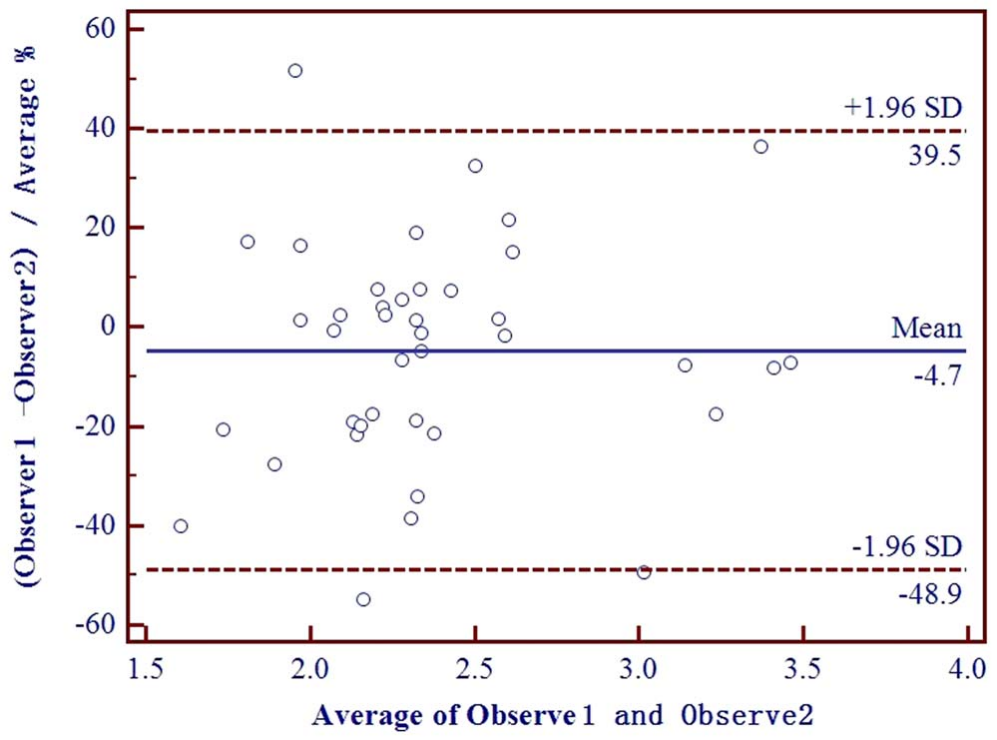
Bland-Altman plot for interobserver SWV in 40 healthy volunteers. The difference of the observers is expressed as percentage deviation from the average of both observers (y axis), and the x axis represents the means of the two observers. The average interobserver difference is represented by the middle solid line, while the limit of agreement is represented by the two outer solid lines. The bias of the two observers is −4.7%, and the limit of agreement is between −48.9% and 39.5%. Two values lie outside the range.

The interobserver agreement, expressed as ICC, was 0.64 (95% CI: 0.13 to 0.82, *P* = 0.011). Interobserver variability was given as a Bland-Altman plot in Figure 3. The bias of the two observers is −4.7%, and the limit of agreement is between −48.9% and 39.5%. Two values lie outside the range. The intraobserver agreement, expressed as ICC, was 0.6 (95% CI: 0.31 to 0.81, *P* = 0.001).

### 2. Potential Influencing Factors

**Figure 4:**
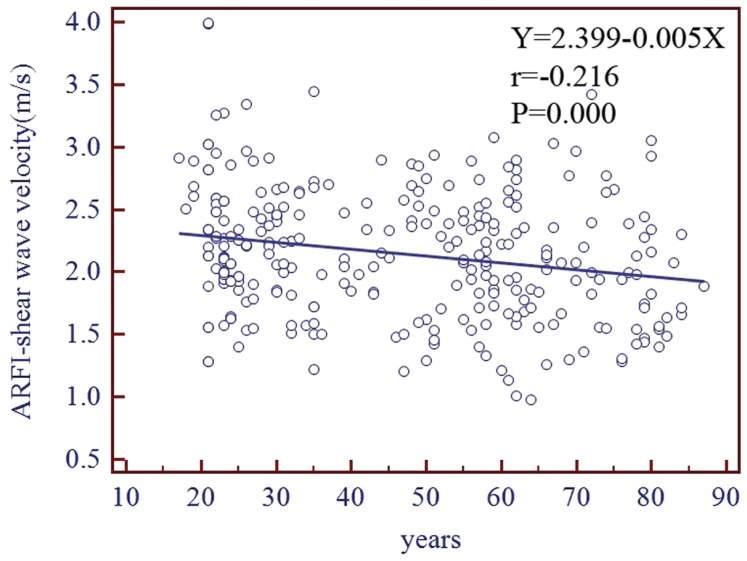
Correlation analysis between SWV and age in 327 healthy volunteers.

Age was correlated significantly with SWV in the 327 healthy volunteers (*r* = −0.22, *P*<0.001) and in the 64 CKD patients (*r* = −0.34, *P* = 0.006). The linear regression equation was *y* = 2.399–0.005*x* in the healthy volunteers, where x and y represented years and the SWV velocity respectively (Figure 4.). SWV differed significantly between men and women in the 327 healthy volunteers (2.06±0.48 m/s vs. 2.2±0.52 m/s, *P* = 0.018) and in the 64 CKD patients (1.55±0.34 m/s vs. 1.9±0.45 m/s, *P* = 0.001). The Pearson’s correlation coefficients analysis showed that SWV did not correlate significantly with height, weight, BMI, waistline, kidney dimension and the depth for SWV measurements (n = 30, from the 327 healthy volunteers). Furthermore, we stratified our study population into two groups according to influencing factors cutoffs, however, there were no significant differences between them (Table 2.).

**Table 2:** Potential influencing factors of SWV in healthy volunteers (n = 30).

**Factors**	**value**	***r***	***P*** a	**groups**	**N**	**SWV**	***P*** b
Height, cm	165.77±7.25 (152–179)^c^	0.089	0.638	≥165	16	2.19±0.37	0.658
				<165	14	2.25±0.39	
Weight, kg	56.33±10.6 (41–77)	0.178	0.347	≥60	9	2.14±0.25	0.455
				<60	21	2.25±0.42	
BMI, kg/m^2^	20.35±2.98 (16.2–29.7)	0.189	0.317	≥24	6	2.17±0.24	0.802
				<24	24	2.22±0.39	
Waistline, cm	75.3±8.01 (62–93)	0.264	0.159	≥80	11	2.19±0.27	0.811
				<80	19	2.23±0.43	
Kidney length, cm	9.9±0.57 (8.84–11.1)	−0.233	0.215	<10	11	2.15±0.35	0.452
				≥10	19	2.25±0.39	
Kidney width, cm	4.59±0.32 (3.91–5.4)	−0.04	0.836	<4.5	19	2.16±0.41	0.3
				≥4.5	11	2.31±0.30	
Depth, cm	4.4±0.66 (3.6–6.07)	0.004	0.985	≥4	21	2.2±0.41	0.733
				<4	9	2.25±0.29	

a Correlation between SWV and variables are analyzed with Pearson’s correlation coefficients,

b Unpaired *t*-test is used to analyze the differences in SWV between two different groups,

c Influencing factors are expressed as Mean ± SD (Range).

### 3. Comparison of SWV between the Healthy Volunteers (n = 327) and the CKD Patients (n = 64)

**Figure 5:**
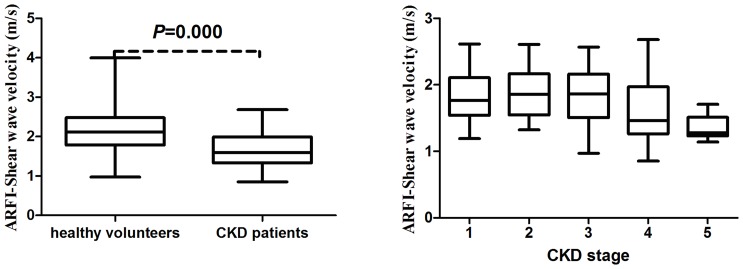
Box and whiskers plot of SWV in different groups. The tops and bottoms of the boxes are the first and third quartiles, respectively. The line through the middle of each box represents the mean. The error bars show the minimum and maximum values (range).

**Figure 6:**
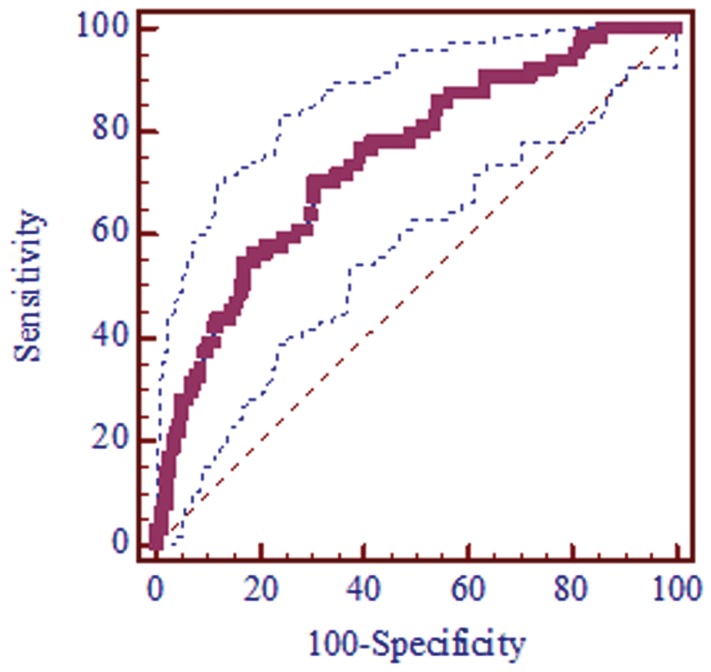
Receiver operating characteristic curves (ROC) estimated the diagnostic performance of ARFI. For a cut-off value of 1.88 m/s, area under the ROC curve: 0.752 (95% CI: 0.704 to 0.797), Standard Error: 0.033, Significance level *P*<0.001. For a cut-off value of 1.88 m/s, ARFI had 71.87% Sensitivity (95%CI: 59.2 to 82.4), 69.69% Specificity (95%CI: 64 to 75), +LR: 2.37,?−LR: 0.4, for predicting CKD.

There was a significant lower mean SWV in CKD patients compared with healthy volunteers (*P*<0.001) (Figure 5). The mean SWVs were 2.15±0.51 m/s (range: 0.97–4 m/s) for the healthy volunteers and 1.69±0.42 m/s (range: 0.85–2.68 m/s) for the CKD patients. For a cut-off value of 1.88 m/s, area under the ROC curve was 0.752 (95% CI: 0.704 to 0.797) (*P*<0.001), the sensitivity and specificity was 71.87% and 69.69% respectively (Figure 6.).

One-way ANOVA showed significant differences in SWV between the healthy volunteers and different stages in CKD patients (*P*<0.001). Multiple comparisons analyzed with the LSD showed that the SWV was significantly higher for healthy volunteers compared with different stages in CKD patients (*P = *0.026, *P = *0.019, *P = *0.003, *P = *0.001, *P*<0.001, in comparison with stage 1, 2, 3, 4, 5, respectively), and it was significantly lower for stage 5 compared with stage 1, 2, 3 and stage 4 in CKD patients (*P = *0.029, *P = *0.037, *P = *0.012, *P = *0.046, respectively). However, there were no significantly differences among stage 1, 2, 3 and stage 4. The mean SWV was 1.81±0.43 m/s (range: 1.19–2.62 m/s), 1.79±0.29 m/s (range: 1.32–2.21 m/s), 1.81±0.44 m/s (range: 0.97–2.57 m/s), 1.64±0.55 m/s (range: 0.85–2.68 m/s), and 1.36±0.17 m/s (range: 1.14–1.71 m/s) for stage 1, 2, 3, 4 and stage 5, respectively, in CKD patients (Figure 5.).

### 4. Comparison of SWV with Laboratory Tests in the 64 CKD Patients

The laboratory data of the CKD patients were shown in Table 3. SWV correlate significantly with e-GFR (*r* = 0.3, *P* = 0.018), serum urea nitrogen (*r* = −0.3, *P* = 0.016) and creatinine (*r* = −0.41, *P* = 0.001) (Table 4.). But SWV did not correlate significantly with serum cystatin C, uric acid, cholesterol, triglycerides, low-density lipoprotein, high-density lipoprotein, calcium, phosphate, hemoglobin, or albumin.

**Table 3:** Characteristics and laboratory data of the 64 CKD patients.

characteristic	CKD1	CKD2	CKD3	CKD4	CKD5
SWV, m/s	1.81±0.43^a^	1.79±0.29	1.81±0.44	1.64±0.55	1.36±0.17
female/male	4/7	7/4	8/12	8/2	10/2
age, year	53.09±16.47	68.09±13.1	68.3±12.58	67.9±16.5	63.67±9.82
eGFR, ml/min/1.73 m^2^	118.87±16.2	73.94±8	47.09±9.24	24.08±5.77	6.56±2.65
cystatin C, mg/l	0.83±0.15	1.15±0.11	1.14±0.36	1.79±0.57	1.9±0.42
urea nitrogen, mmol/l	4.69±0.99	7.28±1.3	8.97±3.27	14.4±3.51	24.38±6.68
creatinine, µmol/l	62.18±12.91	90.73±7.06	127.8±21.22	241.6±50.89	727.17±176.67
uric Acid, µmol/l	313.64±103.96	339.18±70.34	465.8±139.71	615.44±113.95	473±80.89
cholesterol, mmol/l	5.81±2.13	5.55±1.97	5.28±2.74	4.16±1.19	3.94±0.91
triglycerides, mmol/l	1.66±1.08	1.6±0.78	1.64±0.94	1.42±0.74	1.33±0.43
low-density lipoprotein, mmol/l	3.52±1.72	3.37±1.44	3.46±2.21	2.34±0.97	2.32±0.76
high-density lipoprotein, mmol/l	1.31±0.3	1.26±0.5	1.29±0.36	1.14±0.29	0.9±0.14
calcium, mmol/l	2.18±0.13	2.16±0.12	2.2±0.18	2.2±0.24	2.03±0.29
phosphate, mmol/l	1.15±0.23	1.01±0.25	1.11±0.22	1.19±0.27	1.81±0.48
hemoglobin, g/l	131.73±11.98	129.73±15.03	114.95±17.98	103.6±24.88	91.18±19.04
albumin, g/l	38.18±6.66	37.91±6.64	37.45±7.2	39.3±6.45	33.83±5.27

a Variables are expressed as Mean ± SD.

**Table 4:** Correlation of SWV with different variables in the 64 CKD patients.

variable	*R*	*P*
age, year	−0.34	0.006
eGFR, ml/min/1.73 m^2^	0.3	0.018
urea nitrogen, mmol/l	−0.3	0.016
creatinine, mmol/l	−0.41	0.001

## Discussion

Increasing evidences, accrued in the past decades, indicate that the adverse outcomes of CKD, such as kidney failure, cardiovascular disease, and premature death, can be prevented or delayed. Treatment of earlier stages of CKD, irrespective of cause, is effective in slowing the progression toward kidney failure [17]. Recently, increasing awareness of the growing burden of CKD, with a large percentage of the population affected by earlier stages of CKD, has shifted attention and health care priority to the prevention and early detection of CKD. Thus, the introduction of kidney function estimating equations [18] and CKD classifications by the NKF KDOQI (National Kidney Foundation Kidney Disease Outcomes Quality Initiative) [17] and the KDIGO (Kidney Disease: Improving Global Outcomes) [19] have highlighted the condition and facilitated its diagnosis. Hence, nephrologists need sensitive ways to identify CKD as earlier as possible.

However, traditional markers of CKD, such as serum creatinine, urea nitrogen and proteinuria are insensitive and reliance on these may result in extensive time lapse when successful interventions could be tested and applied [20]. Some new biomarkers show promise, such as neutrophil gelatinase-associated lipocalin [21], and some inflammation and fibrotic markers [22], but further validation is required in larger, more diverse populations before translation into clinical practice. So far, it is unlikely that a single marker will satisfy the requirement of CKD progression predicting and CKD early detecting.

Besides serum or urine test, imaging techniques may help to diagnose CKD [23], [24], [25]. Conventional US can provide some effective information to detect ESRD, such as volume reducing, corticomedullary differentiation disappearing, cortical thinning and echogenicity of cortex increased [26], [27], [28]. However, all these descriptive information are subjective, so they can not be quantified according to a universally accepted standard [29]. ARFI, as a quantitative technique differs from conventional US, may have more superiority. Compared with biomarkers mentioned above, ARFI is quite different from them because it is based on biophysical features.

ARFI shows a good positive correlation in evaluation of the grade of hepatic fibrosis and cirrhosis, Kircheis *et al.* [8] reported that SWV increased significantly with the stage of hepatic fibrosis (1.09±0.13 m/s for patients with no significant fibrosis; 1.46±0.27 m/s for patients with significant liver fibrosis; and 2.55±0.77 m/s for patients with liver cirrhosis). Similar conclusion was demonstrated in Bota *et al.*’s evaluation that the cut-off SWV was 1.34 m/s for the diagnosis of significant fibrosis, 1.55 m/s for the diagnosis of severe fibrosis and 1.80 m/s for the diagnosis of liver cirrhosis, respectively [30]. In contrast to that, we found the SWV in healthy volunteers was significantly higher than it in different stages in CKD patients. A negative correlation was likely between the SWV and stage of CKD. The differences of histological changes and mechanical property between kidney and liver may result in this difference.

Furthermore, significant association was observed between SWV and e-GFR, serum urea nitrogen and creatinine. These results support our hypothesis that the change in renal parenchyma stiffness is a useful sign for detecting CKD. However, ARFI could not predict the different stages of CKD except for stage 5, due to overlaps among CKD stage 1, 2, 3 and 4. Studies with larger sample size are mandatory to evaluate whether ARFI is able to make differentiation among them.

The tubulointerstitium comprises 80% of the volume of the kidney. Histological ESRD manifests itself as glomerulosclerosis, vascular sclerosis, and tubulointerstitial fibrosis, with tubulointerstitial fibrosis having consistently been shown to be the best histological predictor of progression [31]. Fibrosis in general tends to increase tissue stiffness [32]. Stock *et al.* [15] described a significant positive moderate correlation between SWV measurements and the grade of fibrosis in renal transplant. Nevertheless, Syversveen *et al*. [33] reported that SWV did not differ significantly in renal transplant with and without fibrosis based on the total 16 measurements of both 2 observers (*P = *0.53 and *P = *0.11 for comparison of fibrosis grade 0 vs. grade 1 and fibrosis grade 0 vs. grade 2/3 respectively), the mean SWV was 2.8 m/s, 2.6 m/s, 2.5 m/s, and 1.8 m/s for grade 0, 1, 2 and grade 3 fibrosis respectively. For observer 1, the SWV was significantly lower for fibrosis grade 2/3 compared with fibrosis grade 0 (*P = *0.02), whereas no such significant difference was found for observer 2 [33]. However, the SWV in CKD patients was significantly lower than it in healthy volunteers in our study. The reason for this remains unclear. Some changes in kidneys of CKD patients other than interstitial fibrosis may have impact on parenchyma stiffness. In the future, the correlation between SWV and pathological change in CKD patient may be able to elucidate the cause of this finding.

The age related decline in GFR in adults is accompanied by pathological findings of glomerulosclerosis and cortical atrophy [17]. The consequences of declining GFR with age have not been carefully studied. It is interesting to find a negative correlation between SWV and age exists in healthy individuals. Lee *et al.* [34] reported that SWV for kidneys changed with age in all children, with the increase most notable in children less than 5 years old. Nevertheless, another study reported that SWV for kidneys did not correlate significantly with age in adults [35]. Therefore, further studies are required to address this topic. The SWV of kidneys in men is lower than it in women, so two series reference ranges may be needed. These remind us that age and gender should be taken into consideration when SWV is used as an indicator for differential diagnosis in CKD. We also tested other potential influencing factors, and found that SWV was not significantly influenced by height, weight, BMI, waistline, kidney dimension (length and width) and the depth for SWV measurements in healthy volunteers. These results indicated that ARFI was able to be used in a wide range, with eliminating the effects of these factors.

As a new technique, several shortcomings should be mentioned. Due to the fixed box dimension (1 cm in length and 0.6 cm in width) of the ROI, this technology does not apply to some CKD patients whose renal parenchyma thickness is less than 1 cm. It has been reported that SWV measurements in renal transplant are dependent on the applied transducer force [36],which could be an important influencing factor and need further evaluation in the future. ARFI in its present stage of development has fair to good inter- and intraobserver agreement in healthy kidneys, despite the fact that repeated measurements were obtained from the same area in kidney parenchyma. Low interobserver agreement was also reported in renal transplant [33]. The sensitivity to movement artifacts and the limited detection depth (maximum, 8 cm) are also the limitations encountered in the application of this new technique.

There are some limitations of this study. First, in our study SWV had high standard deviation in the healthy volunteers and CKD patients. In another study SWV showed highest standard deviation in the kidneys than those in the livers, pancreas, and thyroids [35]. A study in vivo pig kidneys showed that elasticity measurements performed using the supersonic shear wave imaging technique can be influenced by the tissue architecture and intrinsic vascular and urinary pressure [37]. The kidney is a complex organ with the components of blood vessels, renal tubuli and stromal components, thus resulting in widely differing measurements. To decrease it, technology development is needed; more repeated measurements and universal operation standards may be helpful too. Second, because of the relatively small size of CKD cohort, gender and age hadn’t been taken into consideration when SWV was analyzed in CKD patients. The two influencing factors should be carefully considered in further studies with larger patient population. Third, it lacks evaluation of etiologies and pathological changes of CKD patients, which will be taken into our next step of study. Finally, the comparison between conventional US and ARFI, and the combining use of them in detecting CKD need further evaluation.

Although advances in proteomics technologies, sample conditioning, and analysis methods have greatly improved productivity and efficiency in biomarker discovery, biomarker verification and validation remains a significant, costly, and high-risk undertaking in the commercial development and deployment of novel biomarkers for CKD [38]. ARFI, which is based on mechanical property, may be a useful new technology for detecting and classifying CKD potentially. With the improvement of this technology, it may have more advantages and better diagnostic performance in the future.
